# Retinotopic Distribution of Structural and Functional Damages following Bright Light Exposure of Juvenile Rats

**DOI:** 10.1371/journal.pone.0146979

**Published:** 2016-01-19

**Authors:** Anna Polosa, Wenwen Liu, Pierre Lachapelle

**Affiliations:** 1 Department of Neurology and Neurosurgery, McGill University, Montreal, Quebec, Canada; 2 Department of Ophthalmology, Research Institute of the McGill University Health Centre, Montreal, Quebec, Canada; National Eye Institute, UNITED STATES

## Abstract

In the present study, we aimed at better understanding the short (acute) and long term (chronic) degenerative processes characterizing the juvenile rat model of light-induced retinopathy. Electroretinograms, visual evoked potentials (VEP), retinal histology and western blots were obtained from juvenile albino Sprague-Dawley rats at preselected postnatal ages (from P30 to P400) following exposure to 10,000 lux from P14 to P28. Our results show that while immediately following the cessation of exposure, photoreceptor degeneration was concentrated within a well delineated area of the superior retina (i.e. the photoreceptor hole), with time, this hole continued to expand to form an almost photoreceptor-free region covering most of superior-inferior axis. By the end of the first year of life, only few photoreceptors remained in the far periphery of the superior hemiretina. Interestingly, despite a significant impairment of the outer retinal function, the retinal output (VEP) was maintained in the early phase of this retinopathy. Our findings thus suggest that postnatal exposure to a bright luminous environment triggers a degenerative process that continues to impair the retinal structure and function (mostly at the photoreceptor level) long after the cessation of the exposure regimen (more than 1 year documented herein). Given the slow degenerative process triggered following postnatal bright light exposure, we believe that our model represents an attractive alternative (to other more genetic models) to study the pathophysiology of photoreceptor-induced retinal degeneration as well as therapeutic strategies to counteract it.

## Introduction

Chronic exposure to bright light is often considered a potential risk factor for the development and the progression of some human retinal diseases, such as Retinitis Pigmentosa (RP) and Age-related Macular Degeneration (AMD) [1–4]. Rodents exposed to a bright luminous environment will develop a retinal disorder known as Light-Induced Retinopathy (LIR), a condition that was mostly investigated using the adult albino rat as the experimental model [5–10] and which was shown to share some characteristics with the above mentioned human retinopathies [11].

Moreover, in retinal disorders affecting the photoreceptor cells, such as those mentioned above, the pattern of degeneration does not occur symmetrically across the retina. The primary damage can develop either only at the periphery (e.g. RP) or in the central retina (e.g. the macula in AMD) or be localized to specific regions of the retina (e.g. sectorial RP) and then spread towards a preferential direction [12,13]. Consequently, understanding the pathophysiological sequence of events leading to this specific photoreceptor degeneration is of utmost interest as it may help us unveil the mechanisms behind these variations in retinotopic susceptibility to disease process and eventually propose new therapeutic strategies aimed at preventing or limiting the progression of retinal damage. Interestingly, one key feature of the LIR model resides in the asymmetric distribution of the resulting light-induced damage, where the superior-temporal quadrant is the retinal region that always shows the most destruction following bright light exposure [5–7,9,10]. However, it is not yet well understood how these hemiretinal differences develop and progress following a bright light insult.

Previous studies have also shown that age at the time of exposure can significantly influence the severity of the LIR; where younger animals usually exhibit a milder form of LIR even when subjected to more severe (i.e. brighter intensities and/or longer duration) exposure regimens [14–19]. Our previous studies on the juvenile LIR model allowed us to highlight another feature of this acquired retinal degeneration, namely that it proceeds in two successive phases: an acute (during the exposure) and a chronic (following the cessation of light exposure) phase. However, the chronic phase was only briefly documented and how the disease progressed in these still developing animals remained to be elucidated. Understanding what distinguishes the juvenile from the adult LIR, and thus the relationship between retinal maturation and retinal light damage, is of great importance as several of the most debilitating retinopathies (such as RP) often develop early in life.

Consequently, in order to better understand the short (acute) and long term (chronic) consequences of postnatal exposure to a bright luminous environment, structural (histology) and functional (ERGs and VEPs) assessments of the diseased retina were obtained at selected ages (from P30 to P400) following the end of the exposure period (P14-P28). Results obtained strongly suggest that bright light exposure of the juvenile retina triggers a slow degenerative process that is still progressing more than 1 year after the cessation of the exposure regimen resulting in a severely impaired retinal structure and function, thus making the juvenile LIR model an attractive alternative (to other more genetic models) to study the pathophysiology of photoreceptor-induced retinal degeneration.

## Methods

All experiments were conducted in accordance with the ARVO Statement on the Use of Animals in Ophthalmic and Vision Research and were approved by the McGill University-Montréal Children's Hospital Animal Care Committee according to the guidelines of the Canadian Council on Animal Care.

### Light exposure

Light exposure was performed as previously reported by us [18–20]. Briefly, upon eye opening (at P14), juvenile Sprague-Dawley (SD) rat pups (N = 65 pups in total; Charles River Laboratories, St-Constant, Qc, Canada) and their mothers (N = 5 in total) were placed in clear Plexiglas™ cages and exposed (from P14-P28) for 12 hours per day to a bright luminous environment of 10 000 lux (as measured at eye level with a IL 1700 Research light meter; International Light, Newburyport, MA). An equal number of age-matched control pups (N = 65) with their mothers (N = 5) were raised under normal cyclic lighting condition (80 lux, 12h dark/12h light) of the animal care facility.

### ERG recordings

Prior to ERG recordings, the rats were anaesthetised with an intramuscular injection of a mixture of ketamine (85 mg/kg) and xylazine (5 mg/kg). Drops of 1% Mydriacyl Tropicamide were used to dilate the pupils. Throughout the entire duration of the ERG recording, the rats were placed on a homeothermic heating blanket (Harvard Apparatus, Holliston, MA) equipped with a feedback rectal probe fixed at 37°C.

The full field flash ERGs (fERGs) were recorded at 5 day intervals from P30 to P60 and at P90, P120 and P400 (n = 6–8 per group), following the cessation of bright light exposure, using an approach previously described by us [18]. Briefly, following an overnight dark adaptation period of 12 hours, the rats were placed, lying on their left side, in a recording chamber of our design that included a photostimulator (model PS22, Grass Technologies, Warwick, RI, U.S.A.) and a rod desensitizing background light of 30 cd.m^-2^. The ERGs were recorded with a DTL fibre electrode (27/7 X-Static® silver coated conductive nylon yarn, Sauquoit Industries, Scranton, PA, USA) that was placed on the cornea and held in place with a moisturizing solution (Tear-Gel, Novartis Ophthalmic, Novartis Pharmaceuticals Inc, Canada). The reference (Grass E5 disc electrode) and ground (Grass E2 subdermal electrode) electrodes were positioned in the mouth and tail, respectively. Recordings of full-field ERGs (bandwidth: 1–1000 Hz; 10 000 X; 6 db attenuation; Grass P-511 amplifiers) were performed with the Biopac data acquisition system (Biopac MP 100 WS, Biopac System Inc., Goleta, CA, USA). Scotopic ERGs were obtained in response to progressively brighter flashes of white light ranging in intensity from -6.3 log cd.s.m^-2^ to 0.9 log cd.s.m^-2^ in 0.3 log-unit increments [Grass PS-22 photostimulator, interstimulus interval: 10 sec, flash duration 20μs, average of 2–5 flashes depending on intensity]. Photopic ERGs were evoked to flashes of 0.9 log cd.s.m^-2^ (photopic background: 30 cd/m^2^, interstimulus interval: 1 sec, flash duration 20μs, average of 20 flashes). In order to avoid the previously reported light adaptation effect, the photopic recordings were obtained 20 minutes following the opening of the background light [21,22].

Analysis of the flash ERG was also performed as previously reported [18]. In brief, the amplitude of the a-wave was measured from baseline to the most negative trough, while the amplitude of the b-wave was measured form the trough of the a-wave to the most positive peak of the ERG. To evaluate the maximal rod response (rod Vmax), scotopic luminance-response function curves were obtained by plotting, for each animal, b-wave amplitudes against the corresponding flash intensities. A sigmoidal intensity-response regression curve was then applied to fit the data points (Prism 4.0 software; Graph Pad, San Diego, CA). The amplitudes of the a- and b- waves of the mixed rod-cone response (ERG response evoked to the brightest flash delivered in scotopic condition) and the photopic b-wave were also analyzed.

### Multifocal ERG (mfERG) recordings

Prior to mfERG recordings, the rats were anaesthetised [ketamine (85 mg/kg) and xylazine (5 mg/kg); IM] and their pupils dilated (1% Mydriacyl tropicamide). The rats were then placed on a heating blanket (Harvard Apparatus, Holliston, MA) equipped with a feedback rectal probe fixed at 37°C. Multifocal ERGs (mfERGs) (Veris, 5.1 Electro-Diagnosis Imaging, San Mateo, CA) were obtained at seven predetermined experimental time points (P30, P35, P40, P45, P50, P55, P60; N = 3 rats per time points) using a technique previously reported by us [23]. The mfERG stimulus array consisted of 37 hexagons of equal size that alternated between a low (black, 0 cd.m^-2^) and high (white, 200 cd.m^-2^) luminous state. This array was presented against a background of 100 cd.m^-2^ in luminance. Data analysis was limited to the first order kernel only. For each rat, the mfERGs evoked from hexagons projecting to the superior and inferior retinas were added separately to yield an average mfERG response (composite waveform) of the superior and inferior retina respectively. Responses corresponding to the middle row of the mfERG array were included in the composite response of both hemiretinas.

### Visual evoked potential (VEP) recordings

The Visual Evoked Potentials (VEP) was recorded with an active electrode (Grass E2 subdermal electrode) inserted subcutaneously over the occipital cortex (lambda stereotaxic coordinate as per Swanson, 2004 [24]). The ground and the reference electrodes remained in the same location as for the fERG recordings. VEPs (bandwidth: 1–100Hz, 10 000X; 6db attenuation; Grass P-511 amplifiers) were evoked to flashes of 0.9 log cd.s.m^-2^ (interstimulus interval: 1 sec, flash duration 20us, average of 100 flashes; background light at 30 cd.m^-2^). Although up to a maximum of four components could be identified on the VEP tracings [two negative (N1 and N2) and two positive (P1 and P2)], measurements of the amplitude and latency were limited to the most prominent wave (i.e. P2), the amplitude of which being measured from the most negative trough to the peak of P2 and the latency measured from flash onset to peak.

### Retinal histology

The rats (N = 3–7 per group) were perfused [4% paraformaldehyde or 4% glutaraldehyde in PBS buffer (pH 7.4)] under deep anesthesia (Urethane 25% mixture) following which their eyes were enucleated and immersed in 4% paraformaldehyde or 4% glutaraldehyde. In order to ensure proper eye orientation, prior to enucleation a surgical suture was tied on the conjunctiva of the nasal side. Ultra-thin (1.0 μm-thick) sections of the retina were obtained as previously described [18] following which pictures were taken (AxioVison 4.7 software, Carl Zeiss Canada Ltd.). In order to determine the extent of light damage reached at each of the predetermined experimental end points, histological reconstructions of the superior and inferior hemiretinas were obtained by joining together 12–14 consecutive retinal segments of 75μm in width each, which were sectioned at every 340μm from the ONH to the ora serrata. The thickness of the outer nuclear layer (ONL) was measured in each of the 12–14 segments and reported in spidergraph forms. In order to outline the boundaries of the light-damaged area (referred herein as the *photoreceptor hole*), we first identified the retinal segment that showed the maximal damage (i.e. thinnest ONL) and from there, determined at which segment (towards the ONH or ora serrata) the ONL returned to a constant [variation of ±1 row of cell nucleus on subsequent segments] thickness.

### Western Blot

CNTF quantification was performed in normal (n = 12) and LIR (n = 12) retinas harvested at pre-determined time points (P30, P35, P40 and P50; n = 3 per group). The retinas were first bisected along the horizontal meridian passing through the optic nerve head. Then, the superior and inferior hemiretinas were homogenized [sonication at a frequency of 50 Hz (Sonics Vibra Cell, Betatek Inc, Toronto, ON)] separately in a lysis tampon [made of 0.1% sodium dodecyl sulfate, 20 mM Tris (pH 8.0), 135 mM sodium chloride, 1% NP-40, 10% glycerol supplemented with protease inhibitors]. The samples were then cooled for 30 minutes on ice at -4°C and then centrifuged at 13000g for 15 minutes at 4°C, after which the supernatant was collected. Equal quantities of 50μg of each proteic samples were resolved by electrophoresis in a running buffer on 10% sodium dodecyl sulfate polyacrylamide (SDS-PAGE) gel. Proteins were then transferred on a nitrocellulose membrane (Bio-Rad Life Science) and a non-specific blockage in TBST [10 mM Tris (pH 8.0), 150 mM NaCl, 0.2% Tween 20] and 5% lyophilized skim milk was performed for 1 hour at room temperature. Following an overnight incubation with the primary antibody CNTF (monoclonal anti-ciliary neurotrophic factor, clone 4–68, 1mg/ml, Chemicon), the membranes were washed with TBST and subsequently incubated with anti-mouse peroxidase-linked secondary antibodies (Amersham Pharmacia, Baie d’Urfé, Québec, CA). Detection of protein signals was performed using a chemiluminescent reagent (ECL; GE Healthcare, Piscataway, NJ) after which membranes were exposed to an autoradiograph imaging film (X-OMAT; Eastman Kodak, Rocester, NY). The membranes were incubated in a stripping solution (200 mM glycine [pH 2.8], 500 mM NaCl, and 0.7% β-mercaptoethanol) at 55°C for 1 hour, reprobed with an anti y-actin monoclonal antibody (1:8000; Santa Cruz Biotechnology, California, USA) and incubated again with an anti-rabbit peroxidase-linked secondary antibody (Amersham Pharmacia Biotech). Densitometry analysis was performed on scanned autoradiographic films and quantified according to pixel intensity (Quantity One 4.1.0 software; Bio-Rad Laboratories). The densitometry values obtained for the neurotrophic factors were subsequently normalized to the y-actin level in the same blot. A series of three independent Western Blots were performed for each selected group.

### Data analysis

All values are reported as mean ± 1 standard deviation (SD). Statistical significance was determined using a Student’s *t*-test or a one-way factor ANOVA followed by the Tukey post hoc test (Prism 6.0 software; Graph Pad, San Diego, CA) (p<0.05). In order to facilitate the demonstration of regional differences, ONL thicknesses of the superior and the inferior retinas were regrouped in four retinal sectors [Sector: 1–2 (from the ONH to 680μm), 3–5 (from 680 μm to 1700μm), 6–8 (from 1700μm to 3060μm) and 9–14 (from 3060μm to the ora serrata, i.e. ≥ 4760μm)]. The age-dependent rate of ONL loss (in control and light exposed animals) was determined with a linear regression [ONL thickness measured at the four retinal sectors plotted against the corresponding phases of disease progression].

## Results

### The effect of aging on the retinal structure of the normal and light exposed rat: evaluating pan-retinal consequences

**Fig 1:**
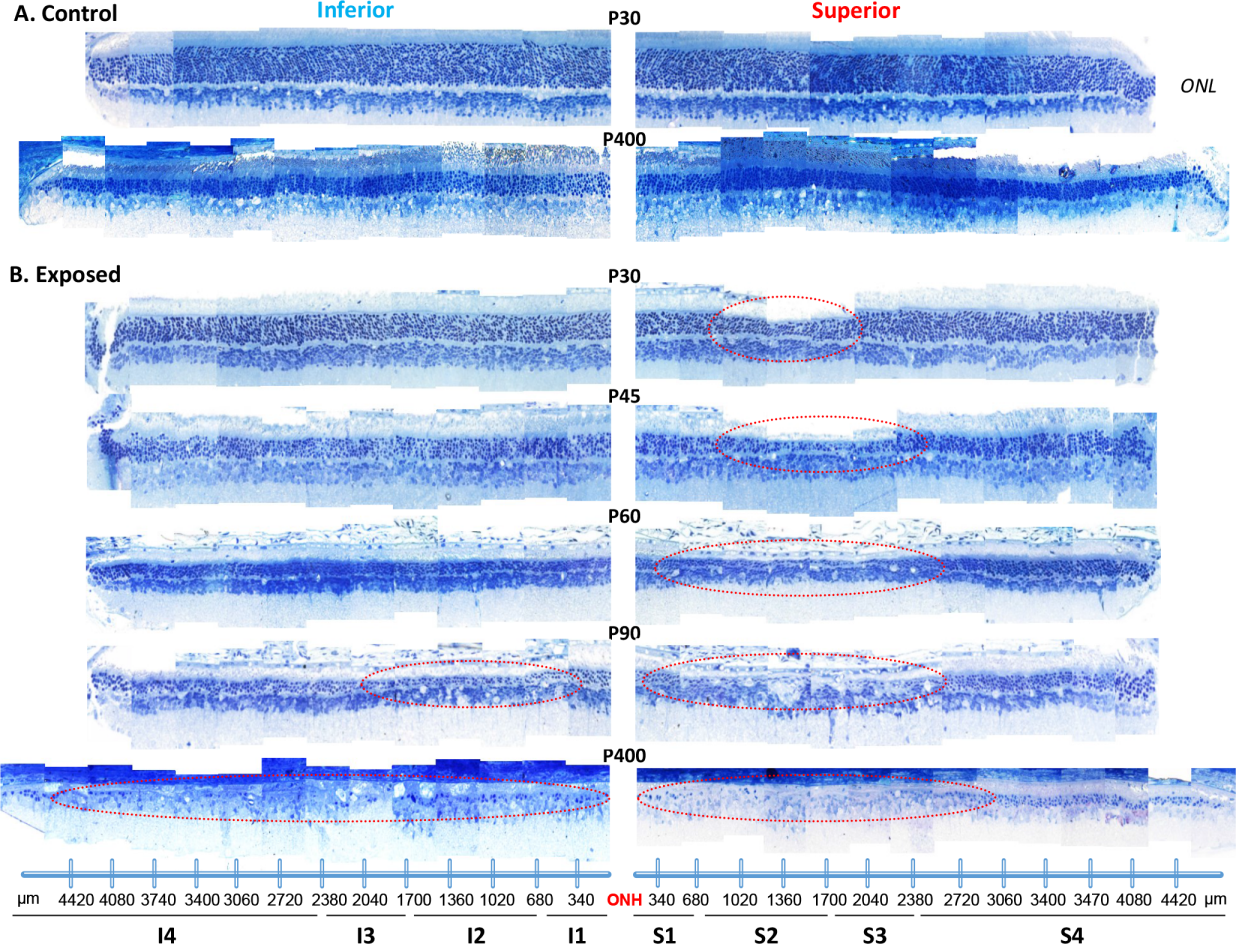
A representative reconstruction of the ONL of the superior (left) and inferior (right) retina (composed of 12–14 consecutive histological segments of 75μm in width, each sectioned at every 340μm from the ONH to the ora serrata for each hemiretina) obtained from control (A) and light exposed (B) juvenile rats at selected postnatal ages [from P30 to P400]. *Abbreviations*: ONL: outer nuclear layer; ONH: optic nerve head; S1 to S4: Sector 1 to 4 for the superior retina; I1 to I4; Sector 1 to 4 for the inferior retina. The extent of the retinal hole is highlighted with red dashed circles in the superior hemiretina.

**Fig 2:**
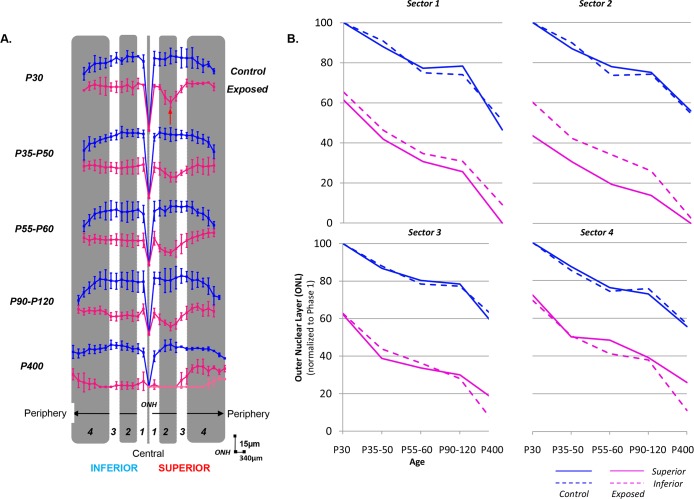
(A) Hemiretinal measurements of ONL loss along the supero-inferior axis in control (blue) and light (pink) exposed rats shown in spidergraph form for different age groups (from P30 to P400). Values were taken at every 340μm from the optic nerve head towards the ora serrata in both the inferior (left) and superior (right) hemiretinas. The red arrow points at the region of maximal damage (i.e. retinal hole). *Abbreviations*: optic nerve head (ONH). Calibration bars: vertical (15μm) and horizontal (340μm). (B) Regional variations in the ONL rate loss. ONL values are regrouped in four different sectors based on the degree of the retinopathy: Sector 1 [from the ONH to 680μm], Sector 2 [680μm-1700μm], Sector 3 [1700μm-3060μm] and Sector 4 [3060μm to 4760μm (ora serrata)] and analysed for each age group in both control (blue) and light (pink) exposed animals for the superior (solid line) and inferior (dashed line) hemiretinas.

In order to determine the extent of the light-induced retinal damage in juvenile rats over time, we performed retinal reconstructions that extended from the ONH to the ora serrata of both superior and inferior hemiretinas. As shown at Fig 1A, for the normal aging rat, despite a gradual, age-dependent thinning of the ONL, one can always appreciate the orderly stacking of the nuclei (ONL and INL) which are uniformly distributed throughout the entire length of the retinal section (e.g. from the ONH to the superior and inferior ora serrata). By P400, the thickness of the ONL is now only 55.20±6.50% of that measured at P30 (best seen with the spidergraphs at Fig 2A). There were no differences noted between the thickness of the superior and inferior ONL, irrespective of retinal eccentricity or age of the rats.

**Fig 3:**
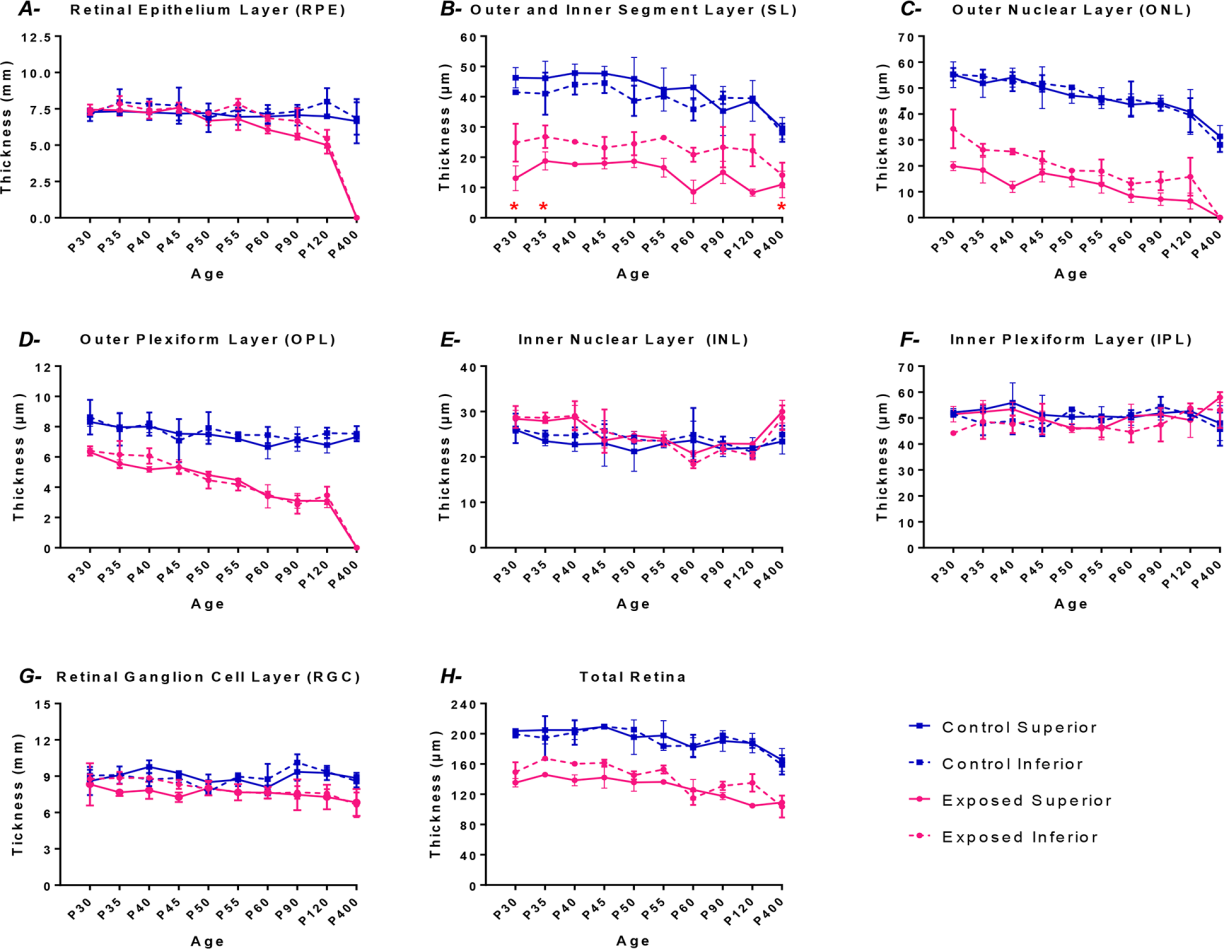
Graphic representation of the thickness (taken at 1000μm from the optic nerve head) of different retinal layers: RPE (A); SL (B); ONL (C); OPL (D); INL (E); IPL (F); RGC (G) and total retina (H) for the control (blue lines) and the light exposed (pink lines) groups in both the superior (solid lines) and the inferior retina (dashed lines). Measurements were obtained at different experimental postnatal periods (from P30 to P400). Results are given as the mean thickness (μm) ± 1SD. Asterisks: Between P30-P35 and at P400, the values reported are those of the thickness of the subretinal space as the length of the outer and inner segments could not be quantifiable (only debris occupied the subretinal space during this period).

Like in normal rats, the retina of light exposed rats also became gradually thinner with age, the thinning being most pronounced within the superior and inferior ONL, as evidenced at Fig 1B. However, as expected, the loss of ONL was most severe in the *“photoreceptor hole area” (*delimited by the dotted red oval at Fig 1B) of the superior retina. At P30, (Figs 1B and 2A), the photoreceptor hole area begins at 607.38±78.34μm (end of sector 1) superior to the ONH and extends to 1727.93±88.72μm (sector 2). There is no evidence of an equivalent thinning of a selective portion of the inferior retina (Figs 1B and 2A), the latter presenting as a relatively uniform layer throughout. Furthermore, as exemplified with the spidergraphs shown at Fig 2A, while in normal the ONL of sector 1 (superior and inferior retina) was thicker than that of sector 4, in LIR both ONL were of equal thicknesses (p>.05). The latter would also suggest a higher susceptibility of the more central ONL (of the superior and inferior retina) to light damage. By P60, the photoreceptor hole within the superior retina had significantly (p < .05) increased in width and now extended between 376.66±86.94μm (first half of sector 1) and 2701.13±163.31μm (beginning of sector 4). At this point, and as exemplified at Fig 1B, the most central portion of this photoreceptor hole only contains a single row of photoreceptor nuclei, compared to 2–3 rows immediately outside of this region and of 4–6 rows in the far periphery [compared to 12 (central) and 9 (periphery) rows of photoreceptor nuclei in age-matched control retinas]. Again, as shown at Fig 1B, at P60, the ONL of the inferior retina is still of uniform thickness (albeit thinner than at P30 or control values) throughout (from the ONH to the ora serrata). Although the size of the photoreceptor hole of the superior retina remained relatively constant between P90 and P120 [P90: 283.15±115.80μm to 2092.97±514.57μm; P120: 397.72±162.01μm to 2364.81±374.49μm; P>.05], it is at P90 that, for the first time, we noticed the emergence of a similar photoreceptor hole in the inferior retina. At P90, the photoreceptor hole of the inferior retina begins at 413.14±103.50μm inferior to the ONH and extends to 2481.12±248.20μm towards the ora serrata and remains more or less of the same size at P120 (244.14±277.75μm to 2358.15±930.77μm, p>.05), a size that is also not different (p>.05) from that of the photoreceptor hole of the superior retina. Expansion of the photoreceptor hole of the superior retina was therefore significantly slower compared to that of the inferior retina. Of note, although the size of the photoreceptors holes, of the superior and inferior retinas, are nearly equivalent between P90-P120, the thickness of inferior ONL is double of that of the superior ONL. The effect of age and light on the ONL thickness (taken from sections at 1000μm superior and inferior to the ONH) is better exemplified at Fig 3C and S1 Fig.

The last samples (taken at P400) revealed further progression towards the near complete destruction of the ONL of the superior retina. As shown in Fig 1B (retina at P400), the photoreceptor hole had grown to cover approximatively 3100±340μm (p<0.05) in width (from ONH towards the ora serrata), representing nearly 60% of the total length of the superior retina (4995.5±109.89μm in P400 control). The remaining ONL of the superior retina (i.e. from the border of the photoreceptor hole to the ora serrata) was composed of 2–3 rows of photoreceptor nuclei (compared to 5–7 in age matched controls). In contrast, it is at P400 that the light-induced damage became significantly more important in the inferior retina. Only a few scattered photoreceptor nuclei appeared to have survived across most of the inferior retina, the width of the damaged zone now covering 4554.79±212.69μm or more than 90% (compared to 75% for the superior retina) of the total length of the inferior retina, an observation identical in all animals. The remaining ONL of the inferior retina was now composed of only 1–2 rows of photoreceptor nuclei (compared to 5–7 in age matched controls and 2–3 in the superior retina of LIR rats). The latter findings were unexpected given that, up to P400, ONL thinning was most important within the superior retina. Finally, considering that in normal rats, the width (from the ONH to ora serrata) of the superior retina is 3835.67±136.68μm (at P30), 4319.08±316.43μm (between P90-120) and 4995.5±109.89μm (at P400), the length of photoreceptor hole (as estimated from the retinal strips) represented approximately 30% of the superior hemiretina at P30, 54% in between P55-60and 75% in P400 (most severe group), compared to approximately 85% with the adult model of LIR.

Of interest, the pattern of ONL attrition not only differed between normal and light-exposed rats as they aged, but also appeared to vary with respect to the retinal sector considered. This is best exemplified at Fig 2B where the thickness of the superior and inferior ONL of normal and light-exposed rats, measured within the 4 different sectors, are plotted against the different group ages of maturation (normal rats) or disease progression (LIR rats). In normal rats, the overall rate of ONL attrition (i.e. from P30 to P400) measured for the superior and inferior retinas are nearly identical; graphs being superposable irrespective of the sectors considered (even the plateau-like effect observed between P55-60 and P90-120). As shown at Table 1, the rate of ONL attrition attributable to the maturation of the superior retina (all sectors considered) significantly slowed down from 1.02±0.04μm/day (measured in the P30 and P35-50 groups) to 0.62±0.14μm/day (P35-50 and P55-60 groups) to 0.07±0.03μm/day (P55-60 and P90-120 groups) to 0.08±0.02μm/day (P90-120 and P400 groups), representing an average 92% reduction (p < .05) in the rate of ONL loss between P30 and P400. A similar (p>.05) rate of ONL attrition was measured within the inferior hemiretina (all sectors considered) between P30 and P35-50 (0.92±0.20-μm/day), P35-50 and P55-60 (0.71±0.47μm/day), P55-60 and P90-120 (0.04±0.01μm/day) and P90-120 and 400 (0.06±0.01μm/day).

**Table 1:** Slope values (representing the rate of ONL loss) obtained from linear regression equations are shown for each group [Control superior (CS); Control inferior (CI); Exposed superior (ES); Exposed inferior (EI)] and sector (1 to 4) analyzed between different age groups: P30 (group 1), P35-50 (group 2), P55-60 (group 3), P90-120 (group 4) and P400 (group 5).

	Sector 1				Sector 2				Sector 3				Sector 4			
Age Group	1 to 2	2 to 3	3 to 4	4 to 5	1 to 2	2 to 3	3 to 4	4 to 5	1 to 2	2 to 3	3 to 4	4 to 5	1 to 2	2 to 3	3 to 4	4 to 5
CS rate	-0.96	-0.71	-0.03	-0.10	-1.05	-0.60	-0.09	-0.06	-1.05	-0.43	-0.06	-0.08	-1.02	-0.74	-0.10	-0.06
CI rate	-0.73	-1.05	-0.03	-0.07	-0.81	-1.08	-0.02	-0.06	-0.96	-0.64	-0.04	-0.06	-1.18	-0.74	-0.05	-0.06
ES rate	-1.57	-0.75	-0.16	-0.08	-1.04	-0.74	-0.17	-0.05	-1.88	-0.34	-0.11	-0.05	-1.77	-0.11	-0.10	-0.04
EI rate	-1.52	-0.78	-0.12	-0.07	-1.44	-0.54	-0.25	-0.08	-1.52	-0.50	-0.25	-0.09	-1.50	-0.64	-0.28	-0.09

A slightly different picture emerges when the data obtained from the light damaged retinas are considered. As exemplified at Fig 2B and Table 1, at P30, light exposure had already significantly reduced the thickness of the ONL to values ranging between 43.61% (Sector 2, superior ONL) and 72.27% (Sector 4, superior ONL) of normal P30 thicknesses (as per the y-intercept values at Fig 2B). This was followed by an additional loss of ONL between P30 and P35-50 groups, the average rate being of 1.57±0.38μm/day for the superior ONL (all sectors considered) and 1.49±0.04μm/day for the inferior ONL (all sectors considered), the two values not being significantly different (p>.05) from each other but significantly (p < .05) faster than those measured in normal. As in normal, this was followed (from P35-50 to P55-60) by a slowing down of ONL attrition to 0.48±0.3μm/day for the superior ONL (all sectors considered) and 0.61±0.12μm/day for the inferior ONL (all sectors considered), representing an average 70% and 60% reduction in attrition rates respectively. The latter values were similar to those measured in normal. Consequently, while the rates of ONL attrition in LIR are nearly similar to normal for sector 1 and, to a lesser extent, for sector 2 as well (except for the fact that the ONL loss is always largest in the superior retina), those measured within sectors 3 and 4 were significantly different from normal (even when considering the presence of a normal-like plateau effect also observed between P55-60 and P90-120 groups). The latter difference was most pronounced between P90-120 and P400 groups in sectors 3 and 4 where the rates of ONL attrition measured for the inferior retina (0.09±0.00μm/day) were significantly faster than those measured for the superior ONL (0.05±0.01μm/day), and significantly faster than the corresponding normal values (0.07±0.01μm/day and 0.06±0.00μm/day, respectively). Taken together, our results would suggest that although at the onset (P30; acute phase) the effects of LIR were most pronounced in the superior ONL, with time (chronic phase) a faster degeneration of the inferior ONL took place.

### The effect of aging on the different retinal layers of the normal and light exposed rat: photoreceptor hole measurements

Apart from the ONL, bright light exposure also caused a significant and gradual thinning of the OSL, ISL and OPL of the superior and inferior retinas, as shown in Fig 3B–3D and S1 Fig. These changes were already present at P30 and persisted throughout, apart from a transient regrowth of the outer and inner segments of the photoreceptors noted between P30 and P40 (Fig 3B). The latter is best visualized in S2 Fig, where a magnification of the outer and inner segments of representative control (top row) and exposed (bottom row) retinas, obtained between P30 and P60, are compared.

Of interest, an equivalent (gradual) thinning of the outer retinal layers was also observed in the normal rats as they aged (Fig 3 and S1 Fig). The above, however, contrasts with the relatively uniform thickness measured for the inner retinal layers (INL+IPL+RGC) of light exposed and control rats as they aged (Fig 3E–3G and S1 Fig). In fact, between P30 and P400, the total thickness of the outer retinal layers (RPE+OSL+ISL+ONL+OPL) of exposed rats was reduced to less than 2% of control compared to 92% of control (p < .05) for the inner retinal layers (INL+IPL+RGC). Similarly, in normal rats, aging (P30-P400) also caused a significantly greater thinning of outer retinal layers compared to inner retinal layers (34.9%±2.8% vs 4.39%±6.76%; p < .05). The latter suggests that, compared to the outer retina, the inner retina is significantly less vulnerable to the detrimental effect of bright light exposure and/or aging. Finally, it is also of interest to note that, in the early phase of the retinopathy (P30 to P40; Fig 3E), the INL of the exposed group was significantly thicker (p < .05) than control, but returned to normal values as the animal aged.

### Assessing the effect of aging on the pan-retinal function of the normal and light exposed rat with the flash ERG

**Fig 4:**
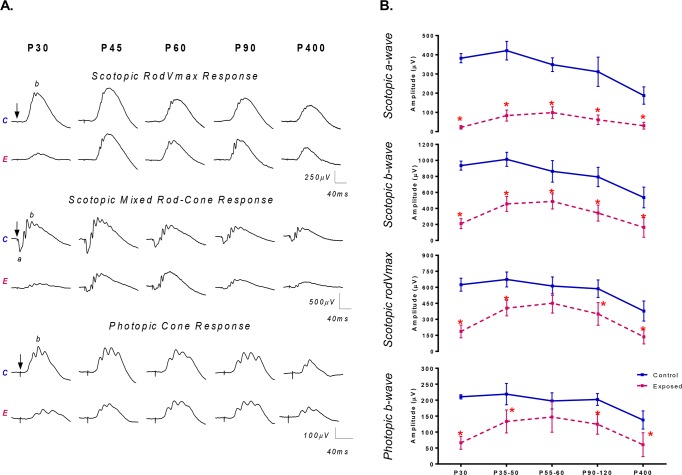
(A) Representative scotopic rodVmax and mixed rod-cone ERG responses and photopic cone ERG responses recorded from both the control (unexposed) and the light exposed animals (P14-28; 10 000lux) at selected experimental time points (from P30 to P400). Horizontal calibration: 40ms; Vertical calibration: 250μV (rodVmax); 500μV (mixed rod-cone); 100μV (cone). A 20ms prestimulus baseline is included in all tracings. Vertical arrows indicate the flash onset. Abbreviations: Control (C); Exposed (E); a-wave (a) and b-wave (b). (B) Graphic representation of the global retinal function (fERG) recorded from different age groups (P30, P35-50, P55-60, P90-120 and P400 groups) of retinal development of normal (control; blue lines) and LIR (exposed; pink lines) rats for the (A) scotopic a-wave; (B) scotopic b-wave; (C) scotopic rodVmax; (D) photopic b-wave. Asterisks represent statistically significant differences (p < .05) compared to control groups. Results are reported as mean 1±SD.

Representative fERG recordings [rod V_max_, mixed rod-cone and photopic responses] obtained from normal (control) and light exposed (P14-28LIR) juvenile albino rats aged P30 to P400 are shown in Fig 4A. Group data is graphically reported in Fig 4B.

In rats raised under a normal lighting environment, all the parameters of the fERG reached maximal amplitudes between P35-P40 as per Fig 4A) of retinal maturation and, with further aging, fERG responses (as per Fig 4A) decreased to reach 46.1%, 37.8% and 38.3% (scotopic a-wave, b-wave and rod V_max_, respectively) and 30.2% (photopic b-wave) of P30 value at age P400 (p < .05) (Fig 4B).

A different pattern of age-dependent ERG changes was observed for juvenile rats that had been exposed to the bright luminous environment (Fig 4A). A severe attenuation of fERG amplitudes was observed immediately following (P30; Fig 4B) the cessation of bright light exposure (a-wave: 5.9%; mix rod-cone b-wave: 22.5%; rod V_max_: 30.0% and photopic b-wave: 31.5% of control; p < .0001). This was then followed by a gradual and significant increase (most pronounced between P30 and P50 as per Fig 4) in amplitude of all fERG parameters to reach maximal amplitude values at P60 [a-wave: 31.8% (p < .001), mix rod-cone b-wave: 56.5% (p < .05), rod V_max_: 73.6% (p < .0001) and photopic b-wave: 74.4% (p>.05) of control]. Of interest, at P60, scotopic rod Vmax and photopic b-wave parameters reached amplitudes that were not significantly different (p>.05) from normal (Fig 4B), while scotopic a- and b-waves reached amplitudes that were significantly (p < .05) lower than normal. With further aging, all 4 ERG parameters declined to reach, at the final time point (P400), amplitudes that remained significantly smaller than normal [scotopic a-wave: 16.3%; mix rod-cone b-wave: 30.3%; rod V_max_: 36.0% and photopic b-wave: 43.8% of control; p < .0001].

### Assessing the effect of aging on the function of the central retina of normal and light exposed rat with the multifocal ERG

**Fig 5:**
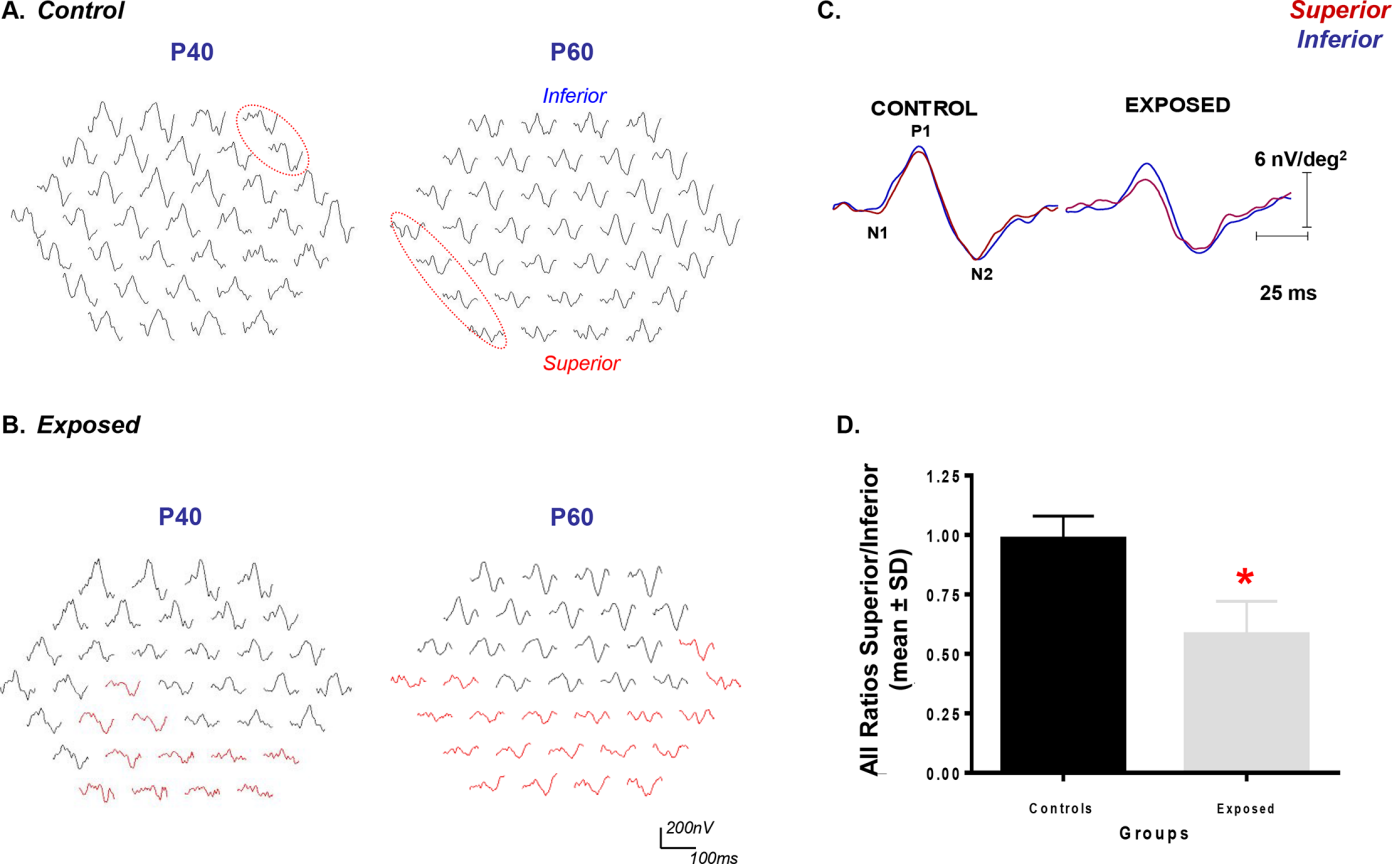
Representative mfERG responses obtained from control (A) and LIR (B) rats at selected postnatal ages (P40 and P60). Tracings are presented in the field view display (i.e. responses from inferior retina at the top; responses of the superior retina at the bottom). Calibration: Horizontal (200nV); Vertical (100ms). In control groups, red circles identify abnormal responses (see text). (C) Composite waves regrouping all the superior (red) and inferior (blue) responses irrespective of age (P30 to P60) in both control and exposed groups. Calibration: Vertical: 6nV/deg^2^; Horizontal: 25ms (D) Quantitative analysis of the summation of all the ratios for the control and exposed animals. (Statistical significance was obtained using t-test, p < .03).

At Fig 5 are shown representative mfERGs obtained from normal (Fig 5A) and light exposed (Fig 5B) rats at selected postnatal ages, as indicated above each mfERG response array. In normal rats, irrespective of the retinal sector stimulated, the mfERG responses are usually of comparable amplitudes and morphologies, with only a few positions (2–4; <10%) showing marked disparities in amplitude and/or morphology. As exemplified at Fig 5A, although these ill-defined mfERGs (surrounded with red dotted ovals) tended to be next to each other, their retinal eccentricity changed randomly as the rats aged, suggesting that these mfERG anomalies were most probably due to methodological limitations rather than suggesting the presence of a developing retinopathy. In fact, the SR/IR ratios were not significantly different from each other (p>.05) and nearly equal to unity, and that, irrespective of age [P40: 1.10±0.28P60: 1.03±0.24, respectively].

In contrast, as exemplified at Fig 5B, mfERG responses obtained from light-exposed rats, especially those evoked from the superior retina, were all significantly reduced compared to control. Furthermore, contrasting with results obtained from normal rats, the abnormal mfERG waveforms were, irrespective of age, consistently most numerous in the inferior hemifield (i.e. corresponding to the superior retina) [SR/IR ratios: P40 (0.47±0.03) and P60 (0.42±0.12); p < .05 from control]. When all the mfERGs waves obtained from the superior and inferior retinas of control and exposed rats (at the 7 time points) were averaged into a single composite waveform (Fig 5C) or when the mean SR/IR ratios measured at each of the 7 time points were averaged to form a single data point (Fig 5D), both showed statistically significant differences between the two hemiretinas of the exposed group compared to normal [Ratio S/I: Control = 0.98±0.10 and Exposed = 0.58±0.14; p < .0001]. The latter further exemplifies the fact that, compared to the inferior retina, the function (and the structure) of the superior retina was significantly more impaired following postnatal exposure to a bright luminous environment.

### Neurotrophic Factor Expression (CNTF)

In a previous study we examined if a change in the expression of some neurotrophic factors (i.e. BDNF, CNTF and FGF-2) could explain the differences in severity noted between the juvenile and the adult forms of LIR [20]. While no changes in the endogenous expression of BDNF were observed between the normal and light exposed (from P14-P20; 6 consecutive days) retinas, a significant up-regulation in both CNTF and FGF-2 levels was noted. Unfortunately, we did not evidence significant hemiretinal differences in the upregulation of these two factors that could have explained the hemiretinal differences noted in the distribution of ONL damage. With this study, we examined if extending the light exposure (from P14 to P28) would enhance the expression of these neurotrophic factors given the significantly more severe LIR. We have, however, decided to limit our investigation to CNTF only for the following reasons: 1- CNTF was previously shown to be preferentially expressed in the outer segments of the photoreceptors (which is where LIR begins) [25], 2- We have previously shown that in the *rds* mouse model, an intraocular gene transfer of CNTF successfully slowed down the progression of the retinal degeneration and enhanced the retinal function as measured with the ERG [26], and 3- Clinical trials are also recognizing the efficacy CNTF supplementation in patients affected with *Retinitis Pigmentosa and Age-related Macular Degeneration*, human retinopathies that are believed to share several features with the animal model of LIR [27–30].

**Fig 6:**
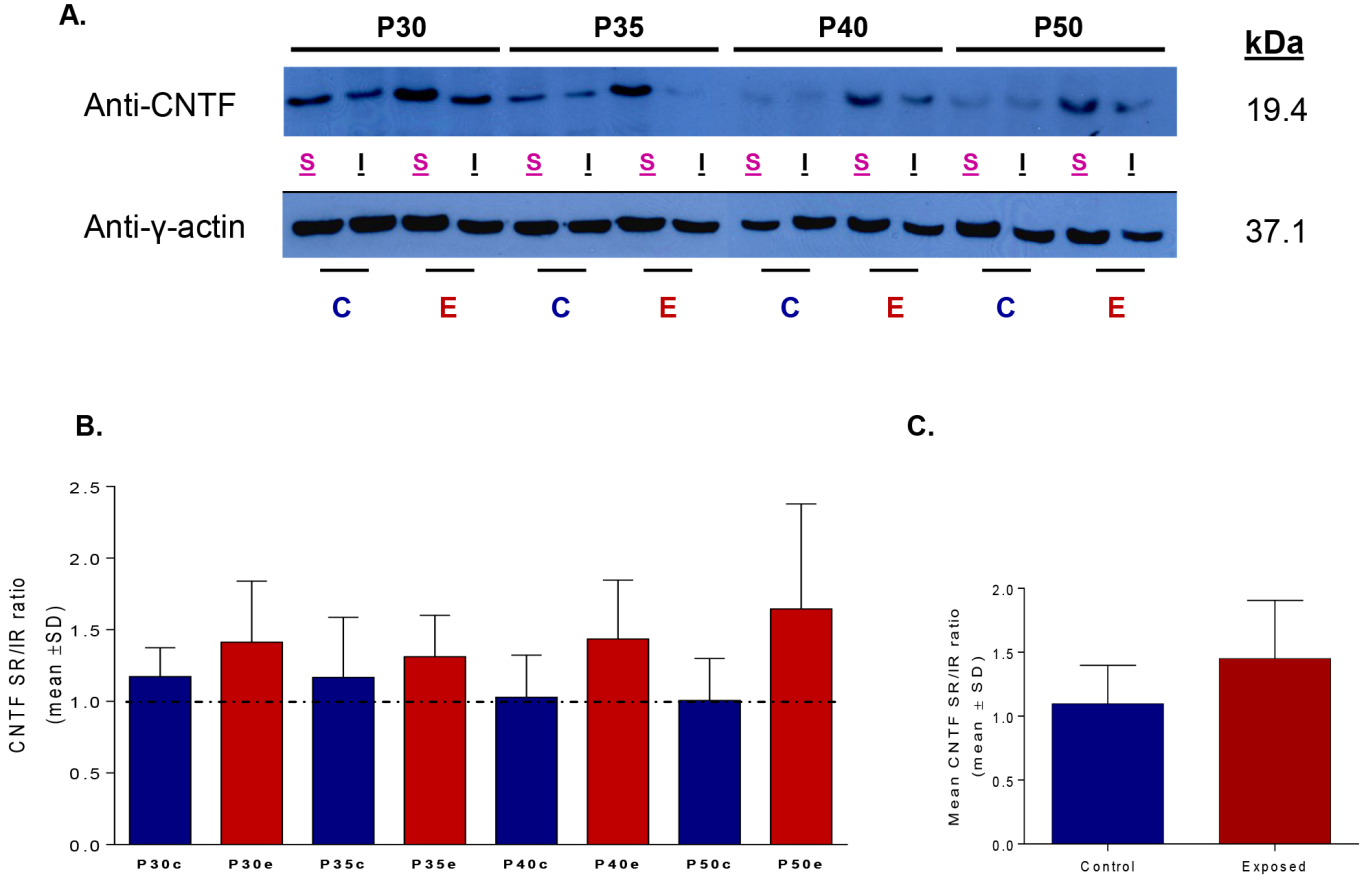
(A) Variations in the expression of the CNTF protein levels in control and light exposed groups collected in the superior and the inferior retinas at selected postnatal days (P30, P35, P40 and P50). CNTF levels were quantified by normalizing its expression with that of the y-actin in each hemiretina. (B) A superior/inferior ratio was then calculated in the control (blue) and light exposed (red) groups to compare CNTF in both hemiretinas. (C) Statistical significance was only obtained when all the control against all the exposed ratios were averaged (1±SD) together (Student’s t-test, p<0.03). Abbreviations: C: control; E: exposed; S: superior; I: inferior. Right: molecular mass (kDa) of CNTF and y-actin.

CNTF protein level expressions measured in the superior and inferior hemiretinas of our control and LIR rats, obtained at selected postnatal days (P30, P35, P40 and P50), are shown in Fig 6A. One can readily appreciate that irrespective of age the highest CNTF levels are always measured in the superior retina of LIR animals. Western blot analysis did not reveal statistical differences in CNTF levels between the superior and inferior retinas of control rats. However, a trend for higher (but not significant, p>.05) CNTF up-regulation was found between the retinas of control and exposed animals [Control mean: 0.54±0.41; Exposed mean: 0.68±0.410, Fig 6B] as well as in the superior retina of exposed rats. The latter resulted in a higher (but non-significant) SR/IR ratio in LIR compared to control rats (Fig 6), irrespective of age [(Control SR/IR ratios at P30: 1.17±0.20, P35: 1.17±0.42, P40: 1.03±0.30, P50: 1.01±0.30); (Exposed) P30: 1.41±0.43, P35: 1.31±0.29, P40: 1.44±0.41, P50: 1.65±0.73]. Significant differences were reached when all (at all age time points; Fig 6C) the data points obtained from the controls (n = 12) and exposed (n = 12) were averaged together [Control mean: 1.10±0.31; Exposed mean: 1.40±0.41] (t-test: *p* < .03).

### Assessing the retinal output of the normal and light exposed rat with the VEP

**Fig 7:**
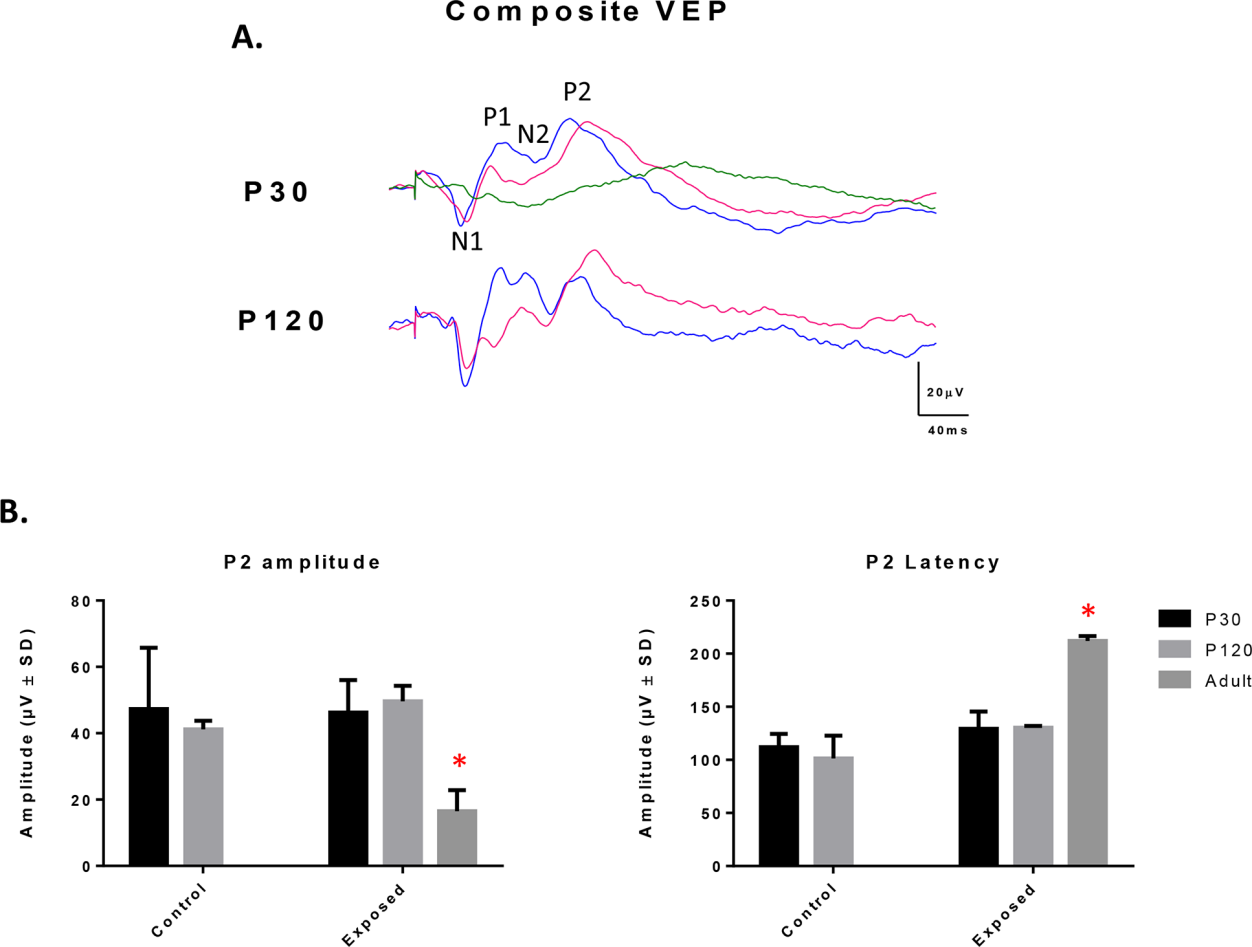
(A) Composite VEP recordings obtained from control (blue) and juvenile light exposed rats (pink) at P30 and P120 and from adult light exposed rats (green) immediately after an exposure of 6 consecutive days (from P60-P66). Vertical calibration bar: 20μV and Horizontal calibration bar: 40ms. Abbreviations: N (negative) and P (positive) components of the VEP. (B) Graphic representation of the amplitude (μV) and peak time (ms) of P2, the most prominent component of the VEP response. Asterisks represent statistically significant differences (p < .05) compared to control groups. Results are reported as mean 1±SD.

At Fig 7A, composite VEP waves (average of 3 rats) recorded from control (blue waves) and juvenile LIR rats (pink waves) at ages P30 and P120 are compared to adult LIR rats (green waves) that were exposed for 6 consecutive days (from P60 to P66). In juvenile rats, there were no significant differences (p>.05) in VEP amplitude between control and exposed rats, irrespective of age (Fig 7B). We did however note a non-significant trend for delayed VEP responses in juvenile LIR rats compared to age-matched controls [P2 peak time at P30: 112.0±12.5 and 129.3±16.3ms, p>.05; and at P120: 101.3±21.6 and 130.3±1.8ms, p>.05; control and LIR rats, respectively] (Fig 7B). In contrast, a significant decrease in amplitude and significant increase in peak time were noted in the adult LIR rat model [16.5±6.4μV and 211.9±4.7ms, respectively; n = 2] compared to control and juvenile LIR rats (Fig 7B).

## Discussion

Our study shows that the pathophysiological manifestation observed during the initial (or acute) phase of the juvenile LIR is concentrated within a well delineated area of the superior retina (i.e. the photoreceptor hole), as previously documented elsewhere [9]. However, during the chronic phase, this photoreceptor hole continues to expand to form by P60, a punched out, almost photoreceptor-free region covering most of superior hemiretina. Of note, while the rate of ONL attrition within the photoreceptor hole (sector 2) followed a control-like (or normal aging-like) decay (1.04 vs 1.05μm/day, respectively), growth of the photoreceptor hole was mainly due to faster rates measured within adjacent regions (sectors 1 and 3; 1.57 and 1.88μm/day, respectively). The resulting hemiretinal asymmetry, which is the hallmark of the rodent LIR, could also be documented functionally with the mfERG (Fig 5). With further aging (between P90 and P120), the degeneration progressively invaded the inferior retina, such that by P400, most of the retina along the superior-inferior axis was now devoid of photoreceptors. Only few photoreceptors remained in the superior far periphery (sectors 3–4). A schematic representation of how we think the juvenile LIR progresses is illustrated in S3 Fig. To our knowledge this is the first demonstration of a progressive, pan-retinal, degeneration resulting from postnatal (or adult) bright light exposure. Whether during the acute phase (i.e. sector 4 has the highest y-intercept values as per Fig 2B) or the chronic phase (sector 4 has the thickest ONL at the end of stage 5 as per Figs 1B and 2B; lowest rate of ONL degeneration is measured in sector 4 between the P90-120 and P400 groups; see Table 1), the peripheral photoreceptors of the superior retina were always the most resistant to degeneration suggesting that they are either structurally or biochemically different or that they are equipped with better defense mechanisms to fight the pathophysiological sequence of events triggered by light exposure. Interestingly, the superior retina was also where the initial ONL loss was first observed. The latter could suggest a retinotopic distribution (or gradient) of photoreceptor susceptibility to disease. Of interest, previous studies [4,31] also reported a relatively better preserved function at the periphery of the superior retina in patients affected with *Retinitis Pigmentosa* (RP), a retinopathy suggested to share common features with animal models of LIR. However unlike our model, in animal models of RP, such as RCS rats or *rds* mice [32,33], the retinal damage starts at the periphery and gradually progresses towards centrally; a pattern of degeneration more reminiscent of the human RP disease process. The greater vulnerability of the central retina seen in our model resembles more the one observed in the *ELOVL4* mice, an animal model of Stargardt’s macular degeneration, where a greater loss of photoreceptors occurs centrally rather than in the periphery [34].

But why is it that the inferior ONL seemed to suffer more from the post-exposure consequences of LIR? Our results showed a non-significant trend for a higher upregulation of CNTF in the superior retina of LIR rats that was maintained during early phases of aging (P30 to P60). When averaged together (P30-P50 values), CNTF upregulation was significantly greater in the superior retina than that of the inferior retina (Fig 6). Presumably the enhanced CNTF production was triggered in response to significantly more severe light-induced damage that was taking place in the superior ONL during the exposure (i.e. from P14-P28), as estimated from the significant difference in the superior-inferior ONL thicknesses measured at P30. Unfortunately, the enhancement in CNTF production was clearly insufficient to prevent the damage from taking place. However given the marked hemiretinal differences in the P30-P60 rate of degeneration between the superior and inferior ONL, one wonders if our demonstration that the superior ONL aged normally between P30 and P60 (compared to a twice faster rate of degeneration measured in the inferior ONL) should not be seen as evidence of a successful CNTF intervention. In fact, given that at P30 the superior and inferior ONL of normal rats were of equal thicknesses (as per Fig 3C), it would be reasonable to assume that, at the onset of light exposure (i.e. P14) they were also of identical thicknesses, that is approximately 55μm. It would then follow that, between P14 to P30, the superior ONL loss 35 μm in thickness (or 2.2 μm/day) compared to 21 μm (or 1.3 μm/day) for the inferior ONL. Interestingly, while between P14 and P30 the superior retina lost nearly twice as much ONL material than the inferior retina, the reverse was observed between P30-P60, the inferior retina now losing nearly twice as much of the ONL compared to the superior ONL. Again, the successful CNTF (and probably other neurotrophic factors) intervention in salvaging the heavily challenged superior retina from fatal degeneration was also evidenced with near 6 fold reduction in the speed of ONL attrition between the estimated P14-P30 (2.2μm/day) and P30-P60 (0.38μm/day) daily losses compared to less than a twofold reduction noted for the inferior retina (1.3 to 0.7 μm/day).

As shown at Fig 4, light exposure initially (measures taken at P30) caused a severe depression of retinal function which was followed by a transient (between P30 and P60) recovery of ERG amplitudes to near normal values (at least for the rod Vmax and photopic b-wave) following which the ERG decreased gradually with age (P90 and P400). This transient increase in ERG amplitudes took place in spite of a gradual degeneration of the photoreceptor layer (Figs 1, 2 and 3), a finding that would appear, on first analysis, counterintuitive. Given that the flash ERG represents the electrical activity of the entire retina, the ERG anomalies measured at P30 would suggest a generalized (panretinal) disorder, the origin of which most probably being a defect in the phototransduction process, as evidenced with the lack of outer and inner segments of the photoreceptors at P30 (lowest ERG responses) and the subsequent gradual growth in ERG amplitudes (Fig 4) along with the re-growth of these segments (S3 Fig). The latter structure-function correlation (i.e. growth in ERG and corresponding growth in outer segments) is reminiscent of the photostasis phenomena previously documented by Penn and Williams (1986) [35]. The limited impact that the initial phases of the juvenile LIR had on the retinal function (2 out of 4 ERG parameters reached normal values by P60 and the other 2 appeared to be still growing) would suggest that the photoreceptor impairment/loss was limited to a relatively small portion of the retina, the size of which being most probably insufficient to cause a severe reduction of all the components of the full field ERG, but sufficient to alter the mfERG responses as evidenced with the results shown at Fig 5. The above contrasted with the structure-function correlations obtained from normal aging rats as well as from the adult LIR model. As shown at Table 2, the ONL thickness (superior+inferior) of normal P400 rats was 55% of the P30 value and the amplitude of the ERG responses (all ERG responses considered), 57.8±6.7% of P30 values. Similarly, the ONL thickness of our juvenile LIR rats aged P400 was less than 6% of normal P30 values (or 11.5% of control age-matched values) while the amplitude of the ERG responses (all ERG responses considered) was 19±9% of P30 values (or 23.5±11.4% of control age-matched values). Similarly, a 6 day exposure (P60-P66) will cause 30 days later (P96) a severe thinning of the ONL to less than 13% of normal P30 thickness accompanied with a severe attenuation of retinal responses (all ERG responses considered) to less than 5% of normal P30 amplitudes (with 2 out of 4 being non-measurable). Consequently, it would appear that the normal aging process equally affected the retinal structure and function (i.e. aging from P30 to P400 similarly reduced the amplitude of the ERG (57.8%) and the thickness of the ONL (55%) for an ONL/ERG ratio of 55/57.8 or 0.95), at least for the age range that we surveyed. Aging of the juvenile LIR rats did not appear to equally affect the retinal structure and function, the ONL/ERG ratio being somewhere between 0.3 and 0.5 depending of the control value considered (P30 or P400). In contrast, aging of the adult form of LIR resulted in an ONL/ERG ratio of 3.0, 30 days following the end of the exposure regimen. Therefore, while the normal aging process equally affected the retinal structure and function, aging of the juvenile LIR rats was more detrimental on the retinal structure while aging of the adult LIR rats was more detrimental on the retinal function. How can we reconcile these two degenerative processes? One explanation could be in how the un-sampled part of the retina reacted to the bright light. It must be remembered that while the flash ERG represents the response of the entire retina, the damaging effect of light exposure on the retina was only sampled over a band of retinal tissue along the superior-inferior axis (passing temporally to the ONH). Consequently, it could be that in the juvenile form of LIR, only a small portion of the retina (adjacent to the sampled area) was damaged by light (explaining the relatively better preserved ERGs), while in the adult form of LIR, most of the retina was lost following bright light exposure (as supported with nearly extinguished flash ERGs) S4 Fig. The latter would point to another difference in the pathophysiology of LIR that would distinguish the adult from the juvenile form and further suggest that they most probably represent two distinct forms of the same disease, clearly a claim needed further investigated.

**Table 2:** Group data comparing the retinal structure and function obtained from normal (at P30, P60, P400), juvenile LIR (at P30, P60, P400) and adult LIR (At P90 or 30 days after end of exposure) rats. Thickness of the outer nuclear layer (ONL; in μm) and amplitudes (in μV) of scotopic (a- wave, b-wave and rodVmax) and photopic (b-wave) ERGs are reported. Mean ± 1 SD.

	Scotopic ERG				Photopic ERG
	ONL	a-wave	b-wave	rodVmax	b-wave
**Normal P30**	53.19±6.03	382.40±23.67	935.29±56.65	625.15±61.03	210.50±6.59
**Normal P60**	39.80±6.43	290.70±66.80	748.60±151.60	612.45±85.65	163.20±38.70
**Normal P400**	29.60±3.46	187.83±45.09	536.99±129.78	377.78±94.12	137.90±28.51
**Juvenile Exposed P30**	29.36±3.46	22.42±9.14	210.49±62.46	187.28±60.23	66.36±20.33
**Juvenile Exposed P60**	16.29±4.94	99.14±30.10	487.82±94.57	450.83±92.25	147.10±47.58
**Juvenile Exposed P400**	3.45±5.67	30.73±17.74	162.67±121.90	135.91±65.98	60.44±37.39
**Adult Exposed D30**	6.59±5.11	0	30.80±5.16	0	9.45±0.79

Finally, as shown at Fig 3E, between P30 and P40, the INL of LIR rats was thicker than that of control, a feature of LIR that we have previously reported elsewhere [18]. Given that it is at the level of the INL that the b-wave is said to be generated, a thicker INL could suggest an increase in b-wave generators in order to magnify the weakened photoreceptor signal thus allowing a relatively normal retinal output in spite of a significantly impaired outer retina. This claim was tested by recording visual evoked potentials (VEPs) in both juvenile and adult LIR rats, the results of which are shown at Fig 7. Although slightly delayed, the retinal output was maintained in the juvenile LIR rats, and that, months after the initial insult. In contrast, VEP signals of adult LIR rats were significantly attenuated and delayed. This relative preservation of inner retinal function, despite a severe destruction of the outer retina, is interesting since it is also a feature observed in some animal models of inherited retinal degeneration, such as RCS rats which were also shown to have a preserved inner retina even in late stages of the disease (6 months) where most of photoreceptors were gone [36]. Similarly, preservation of inner retina is also a feature of some forms of RP in humans [37], a feature of RP that subretinal implant therapy is exploiting.

Collectively, our findings suggest that given the slow rate and the specific pattern of photoreceptor death, our juvenile model of LIR is an attractive alternative (to other more genetic models) to study the pathophysiology of photoreceptor-induced retinal degeneration as well as therapeutic strategies to counteract it.

## Supporting Information

S1 Fig Representative retinal sections of the central superior and inferior retina (taken at 1000μm from the ONH) during the normal retinal development (from P30 to P400) in control (unexposed; A) and following bright light exposure (from P14-28; 10 000lux; B) in albino juvenile rats. Abbreviations: RPE: retinal pigment epithelium, OS: outer segment, IS: inner segment, ONL: outer nuclear layer, OPL: outer plexiform layer, INL: inner nuclear layer, IPL: inner plexiform layer, RGC: retinal ganglion cells, FL: fiber layer. Calibration bar: 75μm.
(TIF)

S2 Fig Magnification of the outer and inner segments of representative control and exposed retinas obtained between P30 and P60. A regrowth of both segments (as indicated with the red arrow) can be observed in the exposed animals following light exposure. Sections are taken in the superior retina at 1000μm from the ONH.
(TIF)

S3 Fig Progression of the photoreceptor holes following bright light exposure of juvenile rats. During light exposure (acute phase), retinal damage is limited to a selective area of the superior-temporal retina, creating a photoreceptor-hole like area. Following the cessation of bright light exposure (chronic phase), this photoreceptor-like area expands progressively, a progression that is initially (P30-P90) limited to the supero-temporal quadrant to invade (at P90) the inferior retina as well. By P400, most of the temporal retina becomes devoid of photoreceptors, except for the far periphery where photoreceptors are relatively well preserved (more in the superior than the inferior retina). Abbreviations: Superior (S), Temporal (T), Inferior (I), Nasal (N), Postnatal day (P).
(TIF)

S4 Fig (A) Retinotopic distribution of retinal damage obtained from representative adult albino Sprague-Dawley rats at 1 and 30 days following an exposure of 6 days to a bright white light of 10 000lux (12h light/12 hours dark). Each retinal slice [representing the superior and inferior retinal quadrants] was reconstructed using 13–14 consecutive histological segments of 75μm in width obtained at every 340μm form the ONH to the ora serrata of each hemiretina. *Abbreviations*: ONH: optic nerve head. Calibration bar: 75μm. The portion of the retina maximally affected by the light exposure is delimited by the red dotted oval. (B) Representative scotopic and photopic ERGs obtained from control and light exposed adult rats. ERGs were recorded after 1, 15 and 30 days following bright light cessation. Vertical calibration bar: Control: 200μV and 50μV; Exposed: 40μV and10μV for the scotopic and photopic ERG, respectively. Horizontal calibration bar: 40ms. A 20ms prestimulus baseline is included in all tracings. Vertical arrows indicate the flash onset. Abbreviations: a-wave (a), b-wave (b) and days (D).
(TIF)
