# Supplementary figures and images for: Retinotopic Distribution of Structural and Functional Damages following Bright Light Exposure of Juvenile Rats
Source: PLoS One. 2016 Jan 19;11(1):e0146979. doi: 10.1371/journal.pone.0146979 (PMC4718541; doi:10.1371/journal.pone.0146979)

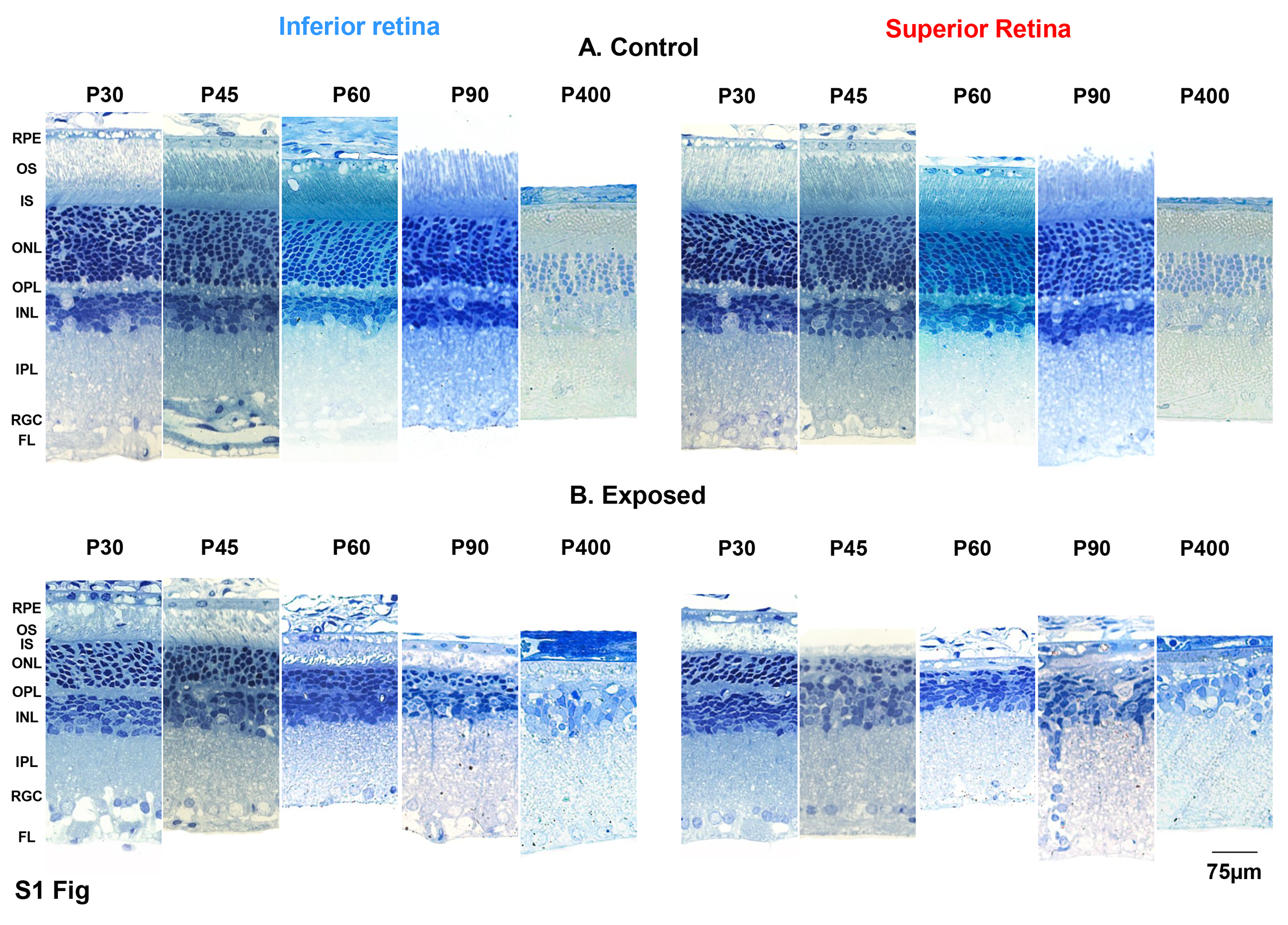

Supplement: S1 Fig — Abbreviations: RPE: retinal pigment epithelium, OS: outer segment, IS: inner segment, ONL: outer nuclear layer, OPL: outer plexiform layer, INL: inner nuclear layer, IPL: inner plexiform layer, RGC: retinal ganglion cells, FL: fiber layer. Calibration bar: 75μm. (TIF) [file pone.0146979.s001.tif]

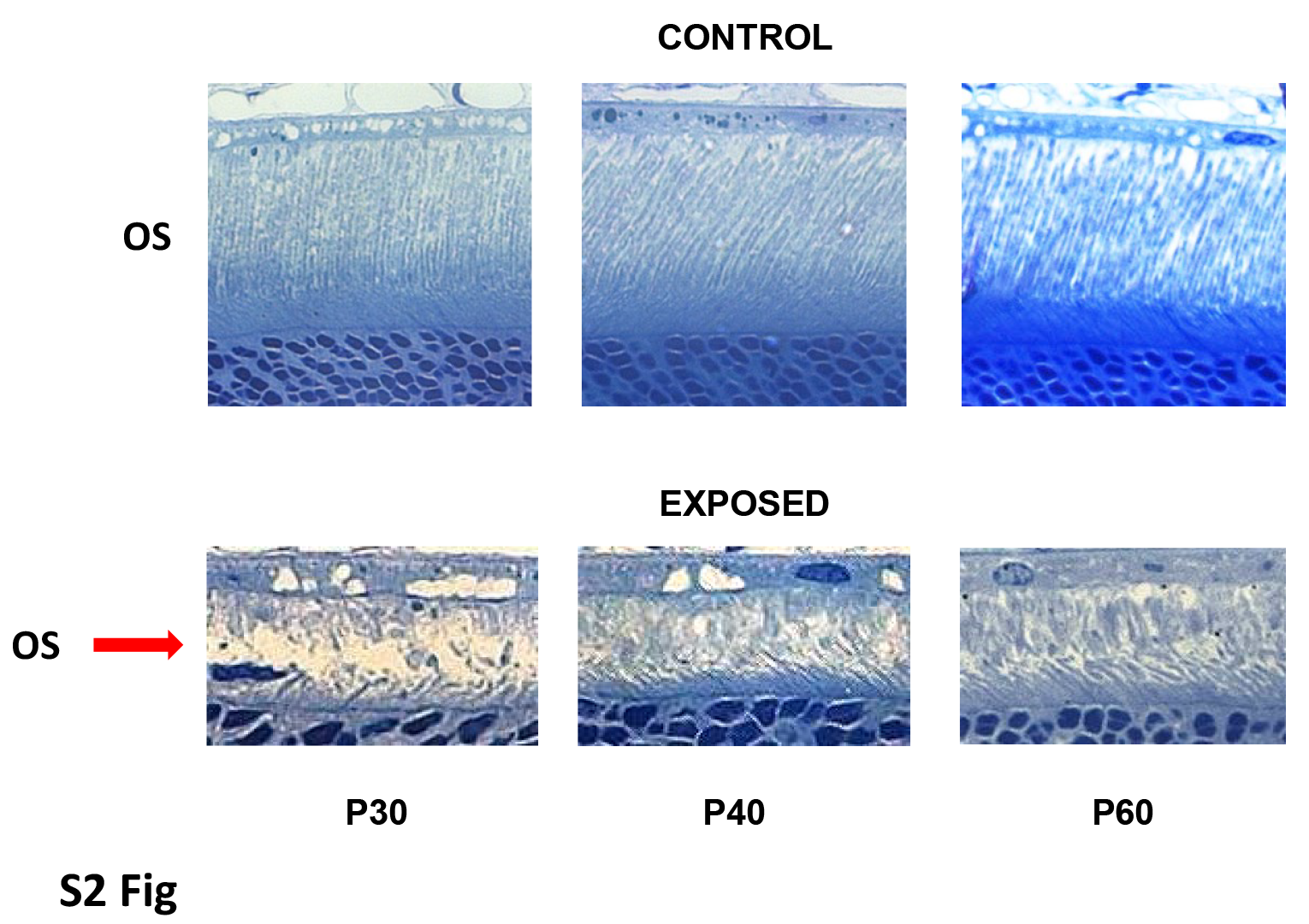

Supplement: S2 Fig — A regrowth of both segments (as indicated with the red arrow) can be observed in the exposed animals following light exposure. Sections are taken in the superior retina at 1000μm from the ONH. (TIF) [file pone.0146979.s002.tif]

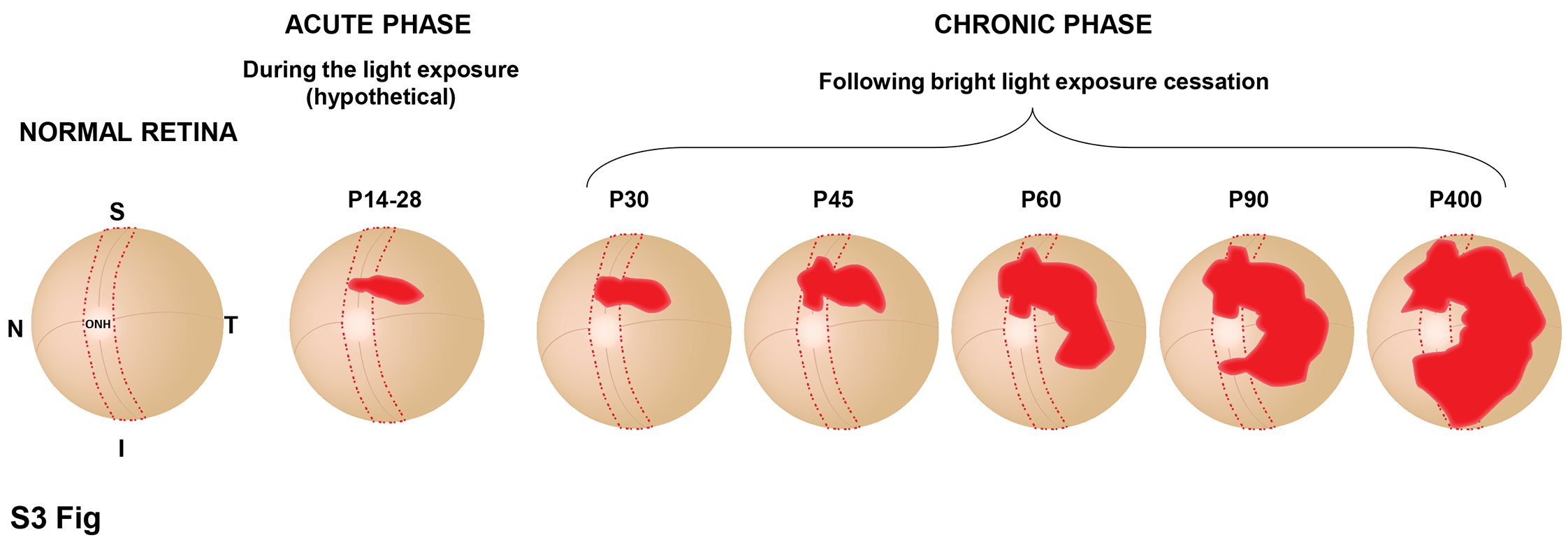

Supplement: S3 Fig — During light exposure (acute phase), retinal damage is limited to a selective area of the superior-temporal retina, creating a photoreceptor-hole like area. Following the cessation of bright light exposure (chronic phase), this photoreceptor-like area expands progressively, a progression that is initially (P30-P90) limited to the supero-temporal quadrant to invade (at P90) the inferior retina as well. By P400, most of the temporal retina becomes devoid of photoreceptors, except for the far periphery where photoreceptors are relatively well preserved (more in the superior than the inferior retina). Abbreviations: Superior (S), Temporal (T), Inferior (I), Nasal (N), Postnatal day (P). (TIF) [file pone.0146979.s003.tif]

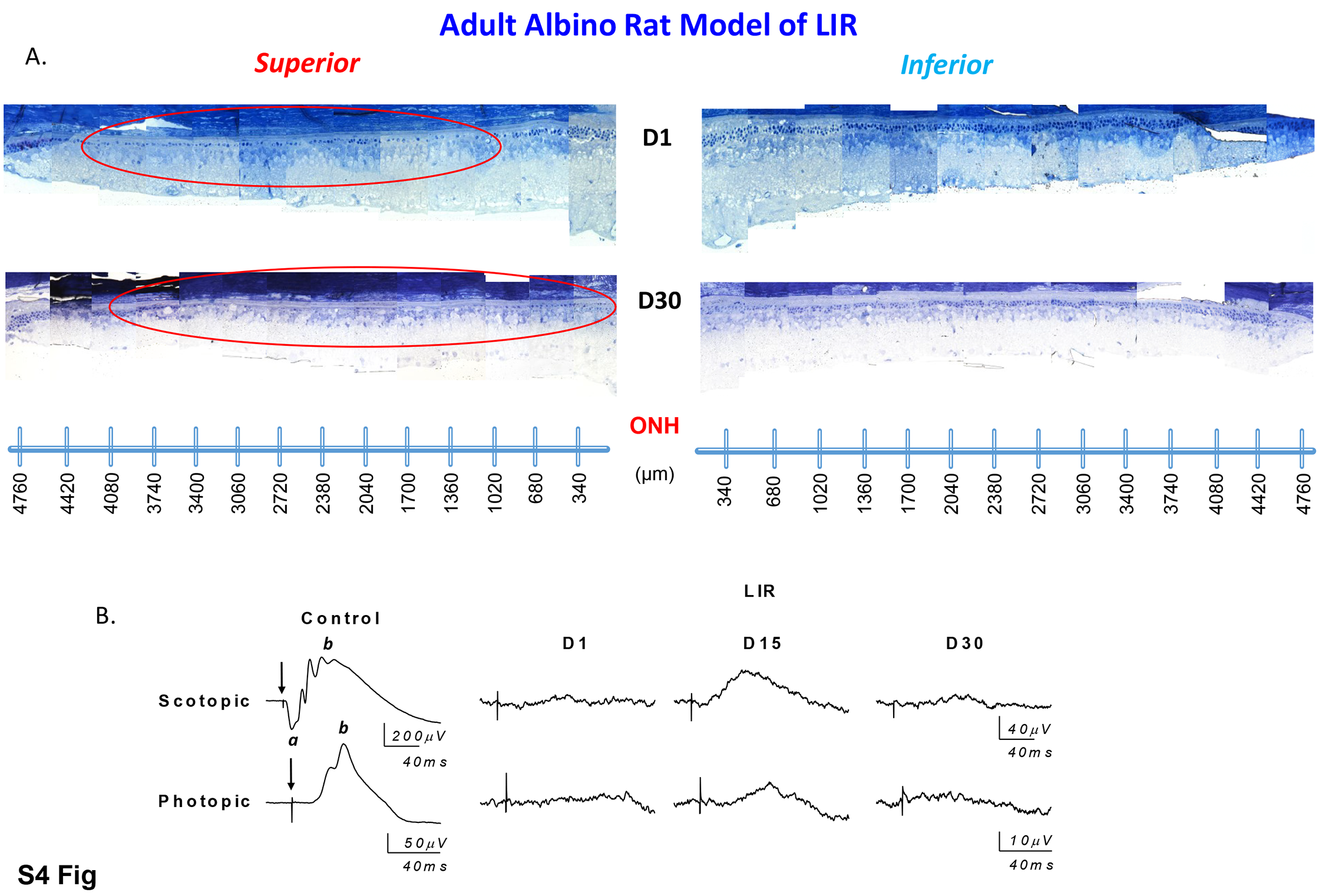

Supplement: S4 Fig — (A) Retinotopic distribution of retinal damage obtained from representative adult albino Sprague-Dawley rats at 1 and 30 days following an exposure of 6 days to a bright white light of 10 000lux (12h light/12 hours dark). Each retinal slice [representing the superior and inferior retinal quadrants] was reconstructed using 13–14 consecutive histological segments of 75μm in width obtained at every 340μm form the ONH to the ora serrata of each hemiretina. Abbreviations: ONH: optic nerve head. Calibration bar: 75μm. The portion of the retina maximally affected by the light exposure is delimited by the red dotted oval. (B) Representative scotopic and photopic ERGs obtained from control and light exposed adult rats. ERGs were recorded after 1, 15 and 30 days following bright light cessation. Vertical calibration bar: Control: 200μV and 50μV; Exposed: 40μV and10μV for the scotopic and photopic ERG, respectively. Horizontal calibration bar: 40ms. A 20ms prestimulus baseline is included in all tracings. Vertical arrows indicate the flash onset. Abbreviations: a-wave (a), b-wave (b) and days (D). (TIF) [file pone.0146979.s004.tif]
